# Identification and validation of CCR5 linking keloid with atopic dermatitis through comprehensive bioinformatics analysis and machine learning

**DOI:** 10.3389/fimmu.2024.1309992

**Published:** 2024-02-27

**Authors:** Bin Zhou, Nuoya Zhou, Yan Liu, Enzhu Dong, Lianqi Peng, Yifei Wang, Liu Yang, Huinan Suo, Juan Tao

**Affiliations:** ^1^ Department of Dermatology, Union Hospital, Tongji Medical College, Huazhong University of Science and Technology (HUST), Wuhan, Hubei, China; ^2^ Hubei Engineering Research Center for Skin Repair and Theranostics, Wuhan, Hubei, China

**Keywords:** keloid, atopic dermatitis, bioinformatics, machine learning, hub genes, immune cell

## Abstract

There is sufficient evidence indicating that keloid is strongly associated with atopic dermatitis (AD) across ethnic groups. However, the molecular mechanism underlying the association is not fully understood. The aim of this study is to discover the underlying mechanism of the association between keloid and AD by integrating comprehensive bioinformatics techniques and machine learning methods. The gene expression profiles of keloid and AD were downloaded from the Gene Expression Omnibus (GEO) database. A total of 449 differentially expressed genes (DEGs) were found to be shared in keloid and AD using the training datasets of GEO (GSE158395 and GSE121212). The hub genes were identified using the protein-protein interaction network and Cytoscape software. 20 of the most significant hub genes were selected, which were mainly involved in the regulation of the inflammatory and immune response. Through two machine learning algorithms of LASSO and SVM-RFE, CCR5 was identified as the most important key gene. Subsequently, upregulated CCR5 gene expression was confirmed in validation GEO datasets (GSE188952 and GSE32924) and clinical samples of keloid and AD. Immune infiltration analysis showed that T helper (Th) 1, 2 and 17 cells were significantly enriched in the microenvironment of both keloid and AD. Positive correlations were found between CCR5 and Th1, Th2 and Th17 cells. Finally, two TFs of CCR5, NR3C2 and YY1, were identified, both of which were downregulated in keloid and AD tissues. Our study firstly reveals that keloid and AD shared common inflammatory and immune pathways. Moreover, CCR5 plays a key role in the pathogenesis association between keloid and AD. The common pathways and key genes may shed light on further mechanism research and targeted therapy, and may provide therapeutic interventions of keloid with AD.

## Introduction

Keloid is a benign fibroproliferative dermal tumor that occurs following abnormal wound healing of skin. It is characterized by excessive myofibroblasts activation and collagen deposition (1–3). It has been found that keloid often progresses and extends beyond the boundaries of the original injury site. Moreover, keloid is not only a cosmetic problem, but also have a significant impact on patients’ psychosomatic health and quality of life. The inflammatory response is often thought to play an important role in keloid formation (4, 5). However, the underlying pathological mechanism of immune cells in the pathogenesis of keloids remains unclear.

It has been reported that keloid is closely associated with atopic dermatitis (AD) in Korean and Taiwanese populations (6). In the case-control study based on the global TriNetX research network, patients with AD have an increased risk of keloid compared to controls without AD (7). Recently, a comprehensive observational analysis of a heterogeneous cohort of UK Biobank participants replicated previously reported disease associations for excessive scarring with eczema, showing a similar trend across ethnic subgroups (Asian, Black and White participants) (8). These findings suggest that AD is strongly associated with keloid. However, the underlying molecular mechanisms explaining the association between the two diseases are complicated and unclear.

AD is the most common and relapsing allergic disease which causing inflammation, redness and irritation of the skin (9–11). It is characterized by a predominant type 2 immune response associated with increased cellular infiltration in the skin, elevated circulating levels of IgE and eosinophilia. In patients with AD, T helper (Th) 2 cells, eosinophils, mast cells and dendritic cells (DCs) are markedly increased in the skin lesions. In addition, Th1 and 17 cells are also involved in the development of AD.

Intensive research is underway to understand the inflammatory mechanisms involved in the development of keloid (12–14). RNA sequencing (RNA-seq) analysis has shown that several Th cell-mediated pathways are significantly upregulated in the microenvironment of keloid, including Th1, Th2, Th17/Th22 and JAK/STAT signaling pathways (15). A variety of inflammatory cells are infiltrated into the microenvironment of keloid lesions, including macrophages, DCs, natural killer (NK) cells, Th cells, T regulatory cells and CD8^+^ T cells. A growing body of evidence suggests that tissue fibrosis is a consequence of an abnormal immune response involving myofibroblast activation and collagen deposition (16). Activation and infiltration of Th cells are thought to be the major cell types leading to fibrosis (17). *In vitro* studies confirmed that type 2 cytokines (IL-4 and IL-13) can increase collagen production in fibroblasts (18). In summary, the above findings support our hypothesis that immune dysregulation, particularly Th cells-mediated pathways, may drive the fibrotic process and link the association between keloid and AD.

In this study, we systematically applied bioinformatics tools and machine learning methods to reveal the common pathways and hub genes underlying the association between keloid and AD. The gene expression datasets of keloid and AD were downloaded from the Gene Expression Omnibus (GEO) database. Machine learning methods were performed to select key genes. Immune cell infiltration and correlation analysis were used to explore the relationship between the key gene and the immune landscape. To the best of our knowledge, this might be the first study to explore the shared gene signatures between keloid and AD. Finally, the key gene CCR5 is identified between keloid and AD, and expected to provide new insights into the common pathogenesis of these two diseases.

## Materials and methods

### Data processing and acquisition

**Figure 1:**
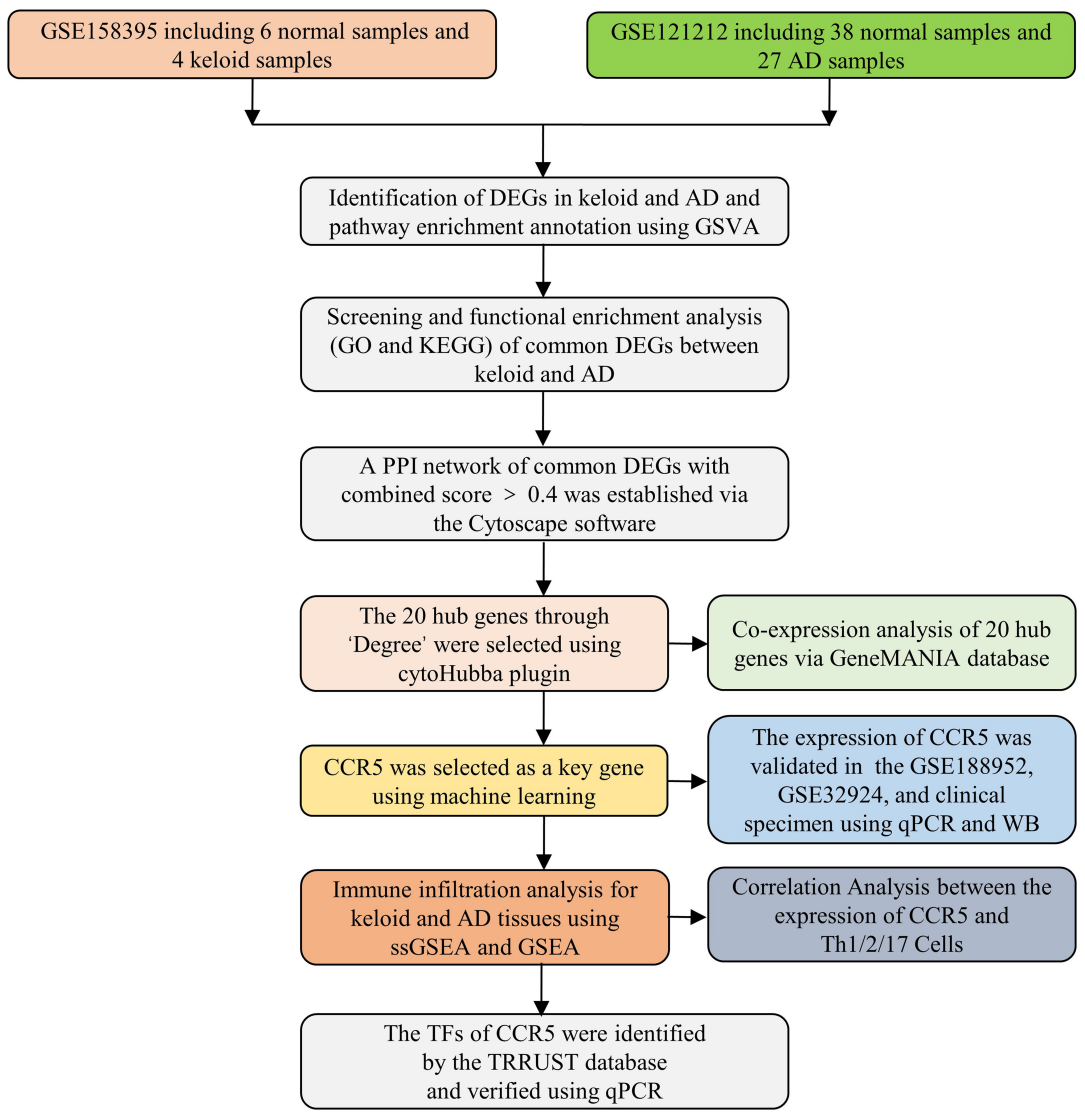
Flow chart of research design.

The gene expression datasets of keloid (GSE158395 and GSE188952) and AD (GSE121212 and GSE32924) were downloaded from the GEO database (https://www.ncbi.nlm.nih.gov/geo/). The gene expression profile data and related annotation files were retrieved. As keloid and AD training datasets, GSE158395 contained 6 normal control samples, 4 lesional and 3 non-lesional samples of keloid patients (19), and GSE121212 contained 38 normal control samples, 27 lesional and 27 non-lesional samples of AD patients (20). As validation datasets, GSE188952 had 3 normal control samples, 4 keloid samples and 5 hypertrophicscar samples. GSE32924 had 8 normal control samples, 13 lesional and 12 non-lesional samples of AD samples (21). We ignored the non-lesional samples because we focused on examining the differences between normal control samples, AD and keloid patients. These datasets had exceptionally good quality control of the data, with complete matrix and clinical information. The probes for these data are publicly available and accessible, and in addition, they contain matrix information that can be well normalized. Standard gene expression normalisation and log2 conversion were performed for the RNA-seq data. The research flowchart of this study is shown in **Figure 1**.

### Differential expression analysis

Differential expression analysis of keloid and AD versus normal control samples was performed using GEOquery and the limma package in R software (22). Screening conditions were adjusted *P* < 0.05 and |log2FC| > 1. Volcano plots and heat maps of differentially expressed genes (DEGs) in the keloid and AD cohorts were generated using the ‘pheatmap’ and ‘ggplot2’ packages. Venn diagram software was used to identify the common DEGs between keloid and AD samples.

### Pathway enrichment analysis

Gene set variation analysis (GSVA) was performed to evaluate pathway enrichment in keloid and AD datasets (23). All hallmark gene sets were downloaded from the Molecular Signature Database (MSigDB) (24). An adjusted *P* value < 0.05 was considered statistically significant. The Benjamini and Hochberg method was used for multiple-testing adjustments.

### Function enrichment analysis of DEGs

To investigate the biological mechanisms of the hub genes linking both keloid and AD, functional enrichment analyses were performed. Gene Ontology (GO) is a database for annotating the functions of genes, including molecular functions, biological pathways, and cellular components. Kyoto Encyclopedia of Genes and Genomes (KEGG) Pathway is a database for the analysis of gene functions and related high-level genomic functional information. The GO plot package and cluster profiler in R were used to analyse the GO function and KEGG pathways to better understand the role of the hub genes (25). The annotation terms with *P* value < 0.05 were considered significantly enriched, and the final results were presented in a bubble diagram and heat map.

### Protein-protein interaction network analysis

Protein-protein interaction (PPI) analysis of DEGs was based on the STRING database (https://cn.string-db.org/), which can search for the relationship between proteins of interest, such as direct binding relationships, or coexisting upstream and downstream regulatory pathways, to construct a PPI network with complex regulatory relationships (26). Interactions with a combined score greater than 0.4 were considered statistically significant. Cytoscape (http://www.cytoscape.org) was used to visualise this PPI network.

### Selection and functional analysis of hub genes

The hub genes (highly connected genes) were selected using the cytoHubba plugin of the Cytoscape software. The selection criteria were set as follows: K-core=2, degree cutoff=2, max depth=100, and node score cutoff=0.2. GeneMANIA (http://www.genemania.org) is a website for building PPI networks, which can be used to generate gene function predictions and locate genes with comparable effects. Physical interaction, co-expression, co-localisation, gene enrichment analysis, genetic interaction and site prediction are some of the bioinformatics methods used by the network integration algorithm. Then a co-expression network of these hub genes was then constructed using GeneMANIA, a reliable tool for identifying internal associations in gene sets (27).

### Selection of key genes through machine learning methods

The LASSO regression and SVM algorithms of machine learning methods can be used to screen the key genes linking keloid and AD (28–30). After an initial filtering of differentially expressed genes, candidate hub genes were identified using two algorithms consisting of LASSO regression and SVM-RFE algorithms. The former was implemented using the “glmnet” package, with the response type set to binomial and alpha set to 1. The latter was performed using the R package “SVM-RFE”, with penalty parameter tuning performed by 10-fold cross-validation and the smallest classification error to determine the variable.

### Validation of key genes expression in validation datasets

The mRNA expression of the identified key genes was verified in validation datasets of keloid (GSE188952) and AD (GSE32924). Comparison between the two datasets was performed using T-test. A *P* value < 0.05 was considered significant.

### Receiver operating characteristic curve analysis

We used the ROC function in the R package to perform ROC analysis. The area under the curve (AUC) of ROC was determined to validate key genes and assess their diagnostic value.

### Analysis of immune cell infiltration

To investigate the different immune cell types in keloid and AD tissues, immune cell infiltration in the microenvironment was assessed using single sample gene set enrichment analysis (ssGSEA) and gene set enrichment analysis (GSEA) software (31, 32). First, the relative proportions of the 28 immune cells in the expression data of GSE158395 and GSE121212 were quantified using the ssGSEA method. These results were presented as a histogram. Functional enrichment analyses of each sample were then performed using GSEA. GSEA analysis of MSigDB gene sets was used for immune infiltration analysis.

### Correlation of key genes with infiltrated immune cells in keloid and AD

To investigate the relationship between identified key genes and infiltrated immune cells, Spearman/Pearson correlation analysis was performed on the gene expression datasets of keloid and AD.

### Prediction and verification of transcription factors

Transcriptional Regulatory Relationships Unraveled by Sentence-based Text mining (TRRUST) is a transcriptional regulatory network prediction database (https://www.grnpedia.org/trrust/) that contains the target genes corresponding to TFs and the regulatory relationships between TFs (33). TFs regulating the hub genes were obtained from the TRRUST database, and adjusted *P* value < 0.05 was considered significant.

### Validation of key genes and TFs in clinical keloid/AD samples

The expression levels of key genes and TFs were verified in clinical samples of keloid and AD. Fresh keloid/AD samples were placed on ice and cut into small tissue pieces. Tissue RNA was extracted using the Trizol method and the concentration of extracted RNA was measured using NanoDrop. The reverse transcription system was prepared, mixed well and added to the PCR instrument for reaction. cDNA after reverse transcription was diluted to 2.5 ng/μL with redistilled water (ddH2O). GAPDH was selected as the internal reference, and primers were designed from the NCBI database, and the primer sequences are shown in **Table 1**. The reaction system was prepared for quantitative real-time PCR (qPCR), and the △△CT method was used for data processing.

**Table 1:** The primer sequences of targets and GAPDH.

Gene	Forward primer (5’ to 3’)	Reverse primer (5’ to 3’)
CCR5	TTCTGGGCTCCCTACAACATT	TTGGTCCAACCTGTTAGAGCTA
NR3C2	GAAAGACGGTGGGGTCAAGTT	ACCGGAAACACAGCTTACGTT
YY1	ACGGCTTCGAGGATCAGATTC	TGACCAGCGTTTGTTCAATGT
GAPDH	TGGCCTTCCGTGTTCCTAC	GAGTTGCTGTTGAAGTCGCA

Tissue protein extraction was performed on ice using RIPA buffer (Sigma-Aldrich, Darmstadt, Germany) containing proteinase and phosphatase inhibitors. Western blot (WB) was performed as previously described (34).

### Ethics statement

The study was reviewed and approved by the Ethics Committee of Wuhan Union Hospital, Huazhong University of Science and Technology. All samples including 6 normal control samples and 6 AD patients; 4 normal control samples and 4 keloid patients were obtained from Wuhan Union Hospital, Huazhong University of Science and Technology after signing written informed consent.

## Results

### Identification of DEGs in keloid and AD

**Figure 2:**
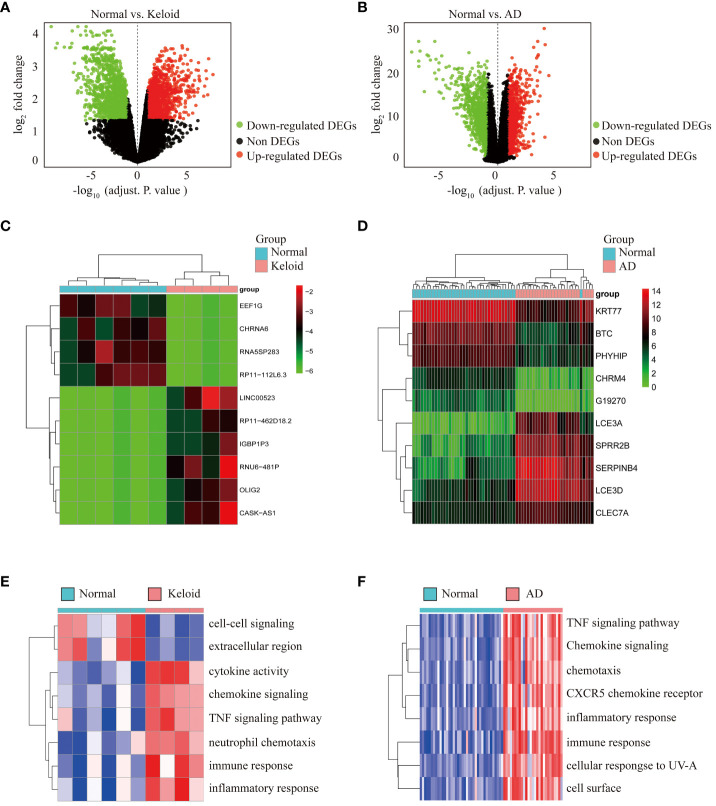
Differential expression analysis and pathway enrichment annotation of keloid and AD. **(A)** Volcano plot showing DEGs between keloid and control groups. Green plots represent downregulated genes and red plots represent upregulated genes. **(B)** Volcano plot showing DEGs between AD and control groups. **(C)** Heatmap showing the top 10 DEGs significantly expressed in keloid samples. **(D)** Heatmap showing the top 10 DEGs in AD samples. **(E)** Heatmap of GSVA enrichment analysis in keloid samples. **(F)** Heatmap of GSVA enrichment analysis in AD samples.

To identify the features associated with keloid, we first obtained 2944 DEGs from GSE158395, including 1443 downregulated genes and 1501 upregulated genes. These DEGs were presented in a volcano plot (**Figure 2A**). Similarly, 2572 DEGs between AD and control groups were obtained from GSE121212, revealing the downregulated expression of 1434 DEGs and upregulated expression of 1138 DEGs (**Figure 2B**). Heat maps of the top 10 DEGs in the gene profiles of keloid and AD were plotted, respectively (**Figures 2C, D**). To further identify the enriched terms in the two datasets, GSVA analysis was performed. The results showed that the gene profiles of keloid were mainly enriched in chemokine signaling, TNF signaling pathway, neutrophil chemotaxis, immune response, inflammatory response, immune-related pathways, etc (**Figure 2E**). The enriched terms of AD were mainly focused on TNF signaling pathway, chemokine signaling, CXCR5 chemokine receptor, inflammatory response, immune response, etc (**Figure 2F**). Therefore, these results showed that both keloid and AD were enriched in TNF signaling pathway, chemokine signaling, inflammatory response, and immune response.

### Functional annotation of common DEGs between keloid and AD

**Figure 3:**
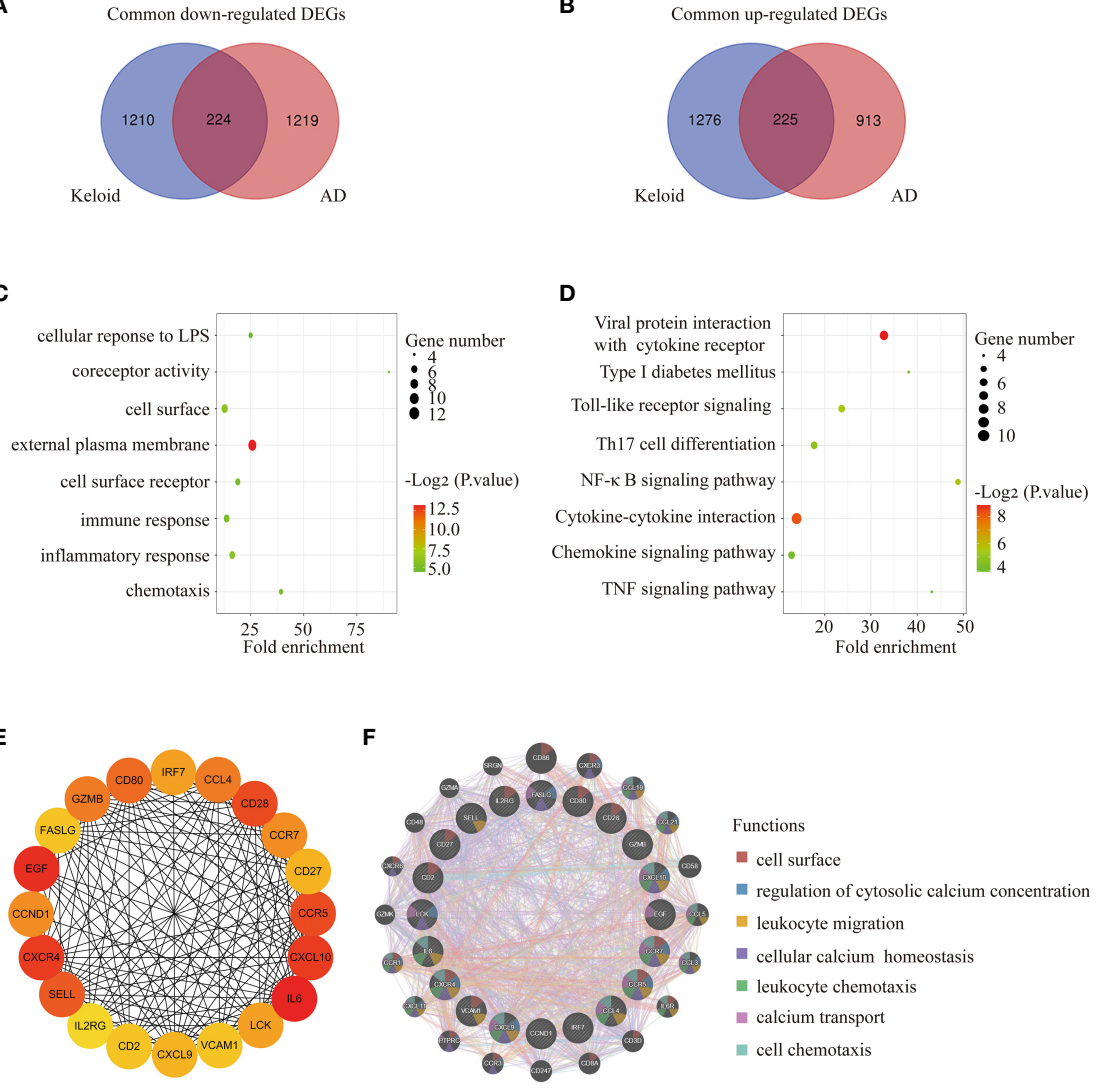
Functional annotation of DEGs and selection of hub genes. **(A)** The Venn diagram shows the intersection of downregulated DEGs from the keloid and AD samples, respectively. **(B)** The Venn diagram shows the intersection of upregulated DEGs obtained from the keloid and AD cohorts, respectively. **(C)** Bubble chart illustrating the significant enrichment terms of co-expressed DEGs in terms of GO enrichment analysis. **(D)** Bubble chart illustrating the significant enrichment terms of co-expressed DEGs in the KEGG analysis. **(E)** The subnetwork of the 20 hub genes with higher degrees in the PPI network selected by MCODE. **(F)** Characterized gene function network of the 20 hub genes.

Venn diagram online tool (http://bioinformatics.psb.ugent.be/webtools/Venn/) was used to identify the common DEGs between keloid and AD. A total of 449 DEGs were found to be shared, with 224 downregulated and 225 upregulated (**Figures 3A, B**). To further understand the function of the co-expressed DEGs, GO/KEGG analysis was performed on the 449 genes. The results of GO analysis showed that the co-expressed DEGs were mainly enriched in external plasma membrane, cell surface receptor, immune response, inflammatory response, chemotaxis, etc (**Figure 3C**). The results of KEGG analysis confirmed that the co-expressed DEGs were closely related to inflammatory pathways, such as Toll-like receptor signaling, Th17 cell differentiation, NF-κB signaling pathway, cytokine-cytokine interaction, chemokine signaling pathway, TNF signaling pathway, etc (**Figure 3D**).

### Selection of hub genes between keloid and AD

The PPI network was constructed using STRING and visualised using Cytoscape. MCODE plugin of Cytoscape was used to identify gene cluster modules with a threshold of combined scores greater than 0.4. 20 hub genes were selected by the Degree algorithm using the cytoHubba plugin. The PPI network of the 20 highly connected genes was visualised using Cytoscape, including 20 nodes and 142 edges (**Figure 3E**). The GeneMANIA database was used to annotate the hub genes. As shown in **Figure 3F**, these 20 genes were associated with leukocyte migration, cellular calcium homeostasis, leukocyte chemotaxis, calcium transport, cell chemotaxis, etc.

### Identification of the key gene CCR5

**Figure 4:**
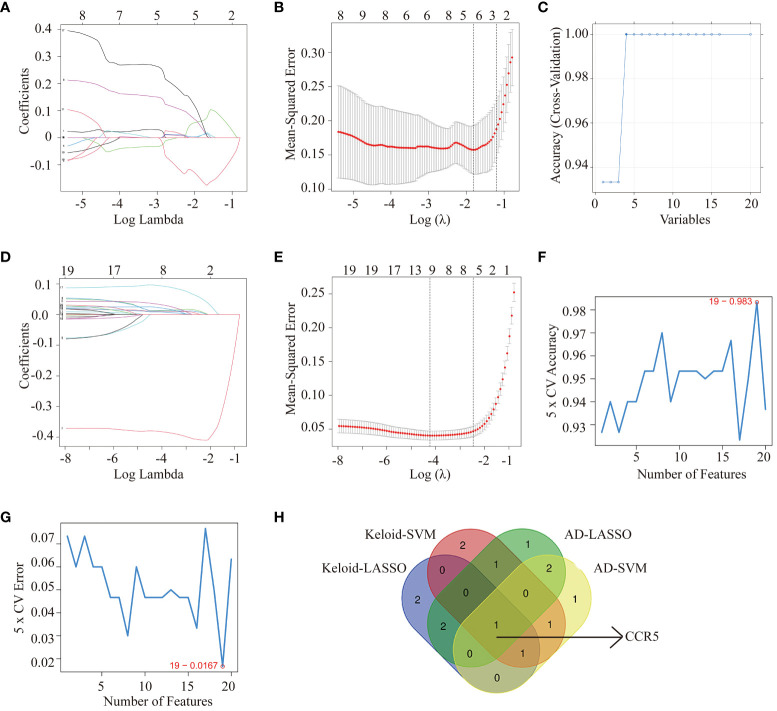
Machine learning methods identifying key genes in keloid and AD. Identification of hub genes for keloid using LASSO analysis **(A, B**) and SVM-RFE algorithms **(C)** from GSE158395. Identification of hub genes for AD using LASSO **(D, E)** and SVM-RFE algorithms **(F, G**) from GSE121212. **(H)** Venn diagram screening overlapping hub genes from LASSO regression analysis and SVM-RFE algorithms in keloid and AD datasets.

We used two machine learning algorithms, i.e. LASSO regression analysis and SVM-RFE algorithms to select key genes. From the keloid dataset (GSE158395) (**Figures 4A–C**), 6 genes were selected by LASSO regression and 6 genes were selected by SVM-RFE. We then screened 7 genes by LASSO regression and 6 genes by SVM-RFE algorithms from AD samples in GSE121212 (**Figures 4D–G**). Venn diagram software was used to identify the common shared key genes between keloid and AD (**Figure 4H**). Finally, the overlapping CCR5 gene was selected as the key gene.

### Validation and testing the diagnostic value of CCR5 gene

**Figure 5:**
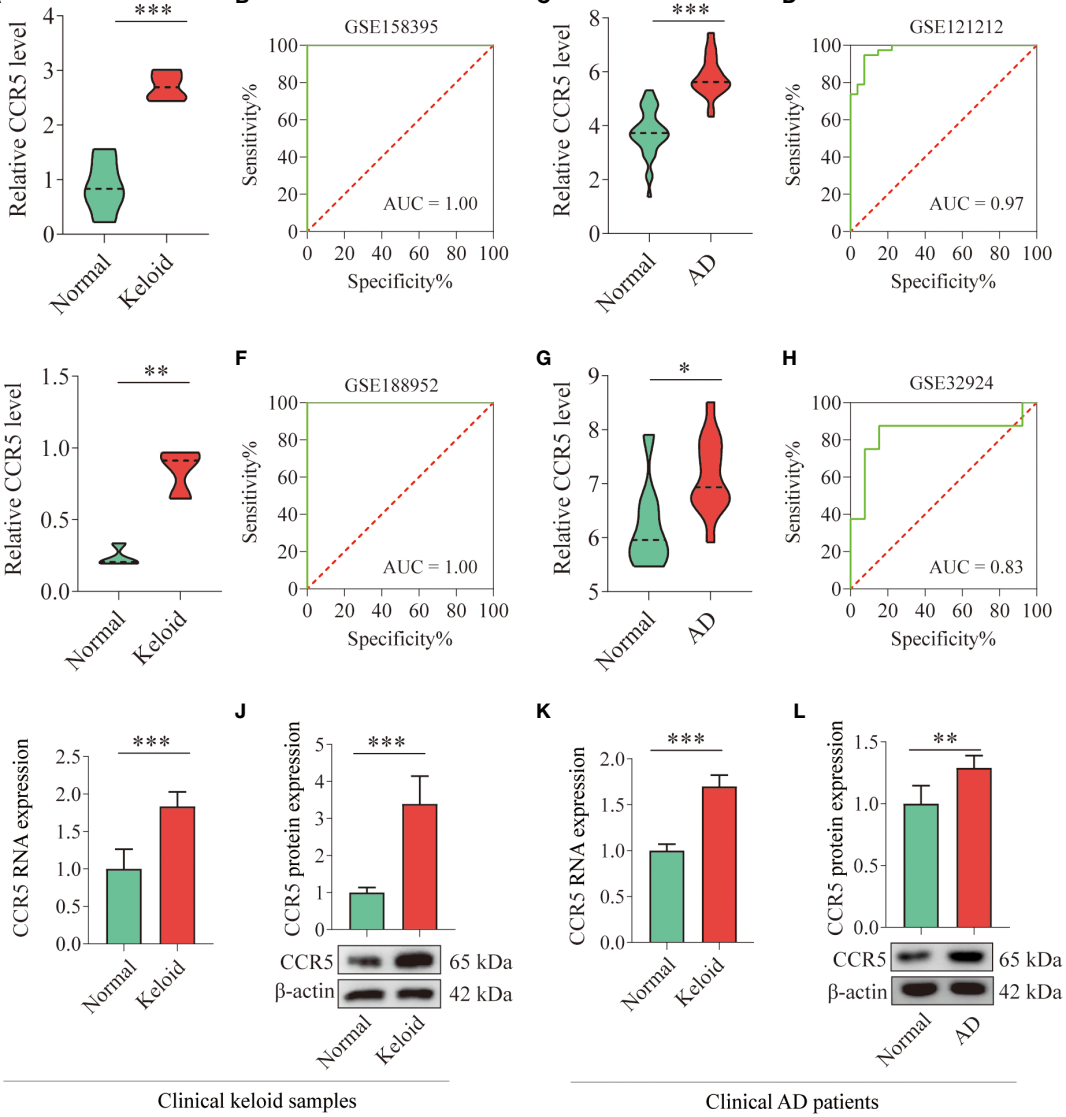
Verification of CCR5 expression in training and validation datasets and clinical samples. **(A)** Histograms showing the expression levels of CCR5 in the gene profile of keloid samples from the training dataset (GSE158395). **(B)** ROC curve analysis of the CCR5 gene in the training dataset. **(C)** Histograms showing the expression levels of CCR5 in the gene profile of AD samples from the training dataset (GSE121212). **(D)** ROC curve analysis of the CCR5 gene in the training dataset. **(E)** Histograms showing the expression levels of CCR5 in the gene profile of keloid samples from the validation dataset (GSE188952). **(F)** ROC curve analysis of the CCR5 gene in the validation dataset. **(G)** Histograms showing the expression levels of CCR5 in the gene profile of AD samples from the validation data set (GSE32924). **(H)** ROC curve analysis of the CCR5 gene in the validation dataset. **(I)** qPCR analysis showing the expression level of CCR5 in clinical keloid samples (N=4). **(J)** WB analysis confirming the expression level of CCR5 in clinical keloid samples (N=4). **(K)** qPCR analysis showing the expression level of CCR5 in clinical AD samples (N=6). **(L)** WB analysis confirming the expression level of CCR5 in clinical AD samples (N=6). * *P* < 0.05, ** *P* < 0.01, *** *P* < 0.001.

The expression of CCR5 was highly expressed in the gene profiles of both keloid (*P*<0.001) and AD (*P*<0.001) in the training datasets (**Figures 5A, C**). The ROC curve suggested that the AUC values of CCR5 in the keloid and AD training datasets were 1.00 and 0.97, respectively (**Figures 5B, D**). To further verify the diagnostic value of CCR5, we examined the expression of the CCR5 gene in the validation datasets and clinical samples. Consistent with the findings in the training datasets, the expression of CCR5 was highly expressed in the gene profiles of both keloid (*P*<0.01) and AD (*P*<0.05) samples from the validation datasets (GSE188952 and GSE32924, **Figures 5E, G**). The AUC values in the validation dataset also confirmed the results: AUC of keloid is 1.00 and AD is 0.83 (**Figures 5F, H**). We then verified the expression of the CCR5 gene using qPCR and WB analysis and repeated three times respectively in 6 normal control samples and 6 AD patients; 4 normal control samples and 4 keloid patients. The results showed that the expression of CCR5 is higher in keloid/AD samples than in the healthy control group (**Figures 5I–L**). Therefore, these results suggested that CCR5 was involved as an important key gene and has excellent diagnostic value in both keloid and AD.

### Analysis of immune cell infiltration in keloid and AD

**Figure 6:**
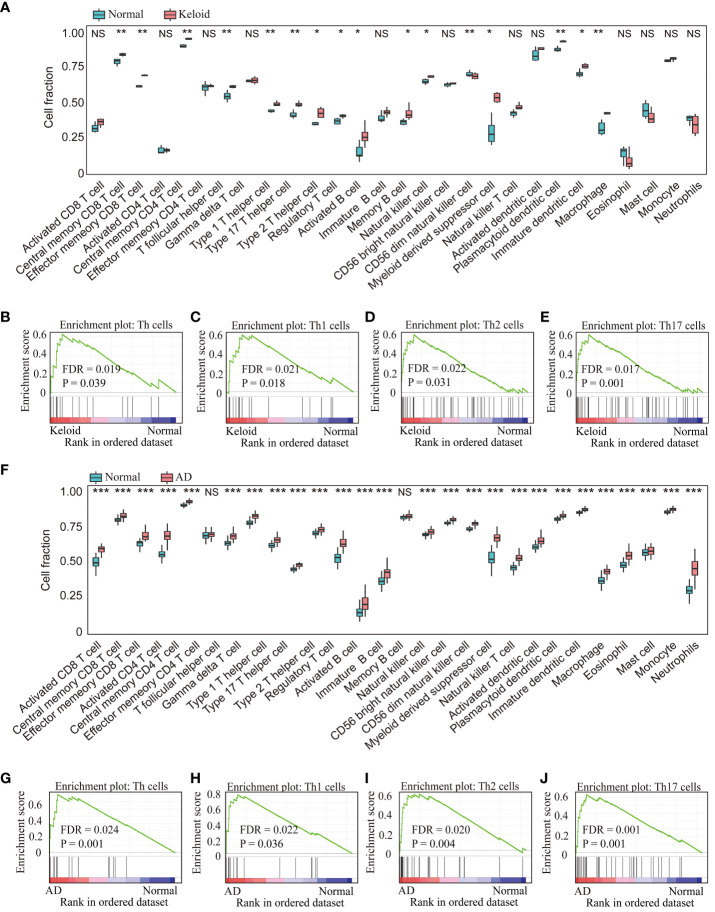
Analysis of immune cell infiltration in the keloid and AD datasets. **(A)** The relative percentage of 28 immune cells in each sample of the keloid dataset (GSE158395). GSEA analysis revealed the enriched cells in the samples of the keloid dataset: **(B)** Th cells, **(C)** Th1 cells, **(D)** Th2 cells, **(E)** Th17 cells. **(F)** The relative percentage of 28 immune cells in each sample of the AD dataset (GSE121212). GSEA analysis revealed the enriched cells in the samples of the AD dataset: **(G)** Th cell, **(H)** Th1 cells, **(I)** Th2 cells, **(J)** Th17 cells. * *P* < 0.05, ** *P* < 0.01, *** *P* < 0.001. NS: no significance.

Immune cell infiltration was assessed using ssGSEA and GSEA, which contains 28 human immune cells. First, gene expression data from GSE158395 was used to quantify the relative proportions of the 28 immune cells in keloid tissue. As shown in **Figure 6A**, the keloid tissue has more immune cell infiltration than the control group, including memory CD4/8^+^ T cells, Type 1/2/17 helper cells, dendritic cells, macrophages, etc. GSEA analysis further showed that the keloid tissues were enriched in Th cells (FDR=0.019, *P*=0.039), Th1 cells (FDR=0.021, *P*=0.018), Th2 cells (FDR=0.022, *P*=0.031) and Th17 cells (FDR=0.017, *P*=0.001) pathways (**Figures 6B**, **2E**). Then the immune infiltration of AD tissues was identified using the gene expression data from GSE121212. Similarly, various types of immune cells infiltrated the AD tissues, including CD4/8^+^ T cells, memory CD4/8^+^ T cells, type 1/2/17 helper cells, dendritic cells, NK/NKT cells, macrophages, etc (**Figure 6F**). The most upregulated immune infiltration terms in AD tissues were enriched in the Th (FDR=0.024, *P*=0.001), Th1 cells (FDR=0.022, *P*=0.036), Th2 cells (FDR=0.020, *P*=0.004) and Th17 cells (FDR=0.001, *P*=0.001) pathways (**Figures 6G–J**).

The similar results were confirmed in the GSE188952 and GSE32924 (**Supplementary Figure 1**). In addition, the factors related to Th1, Th2, and Th17 cells such as IL4/IL13, IL17A/IL22, ADAM33, IFN-γwere upregulated overall in keloid and AD (**Supplementary Figure 2**).

### Correlation analysis between the expression of CCR<n5o></no> and Th1/2/17 cells

**Figure 7:**
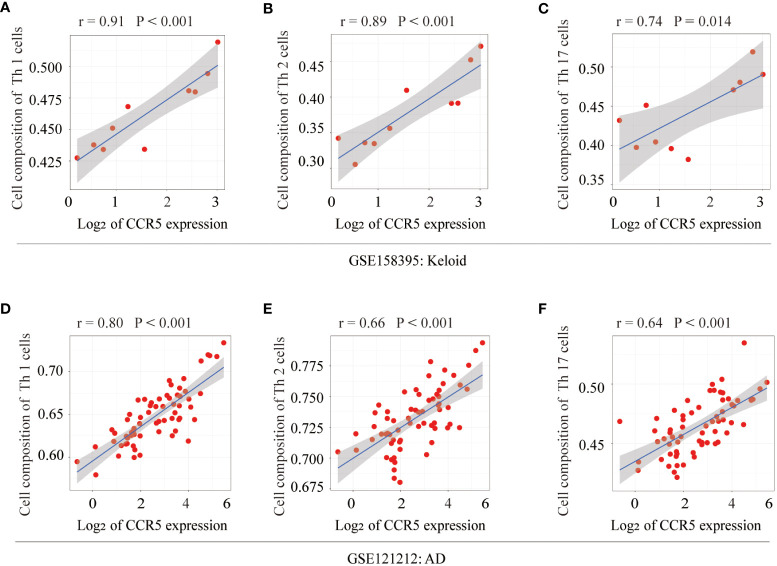
Correlation analysis of the key gene CCR5 with Th cells. In keloid tissue, correlation analysis and scatterplot of CCR5 expression levels with **(A)** Th1 (r=0.91, *P*<0.001), **(B)** Th2 (r=0.89, *P*<0.001), **(C)** Th17 cells (r=0.74, *P*=0.014). In AD tissue, correlation analysis and scatter plot of CCR5 expression levels with **(D)** Th1 (r=0.80, *P*<0.001), **(E)** Th2 (r=0.66, *P*<0.001), **(F)** Th17 cells (r=0.64, *P*<0.001).

The relationship between CCR5 expression levels and immune cell abundance was analyzed, which showed that CCR5 was positively correlated with Th1 (r=0.91, *P*<0.001), Th2 (r=0.89, *P*<0.001) and Th17 cells (r=0.74, *P*=0.014) in keloid samples from GSE158395 (**Figures 7A–C**). In addition, it is worth noting that the expression level of CCR5 also had an ideal correlationship with Th cells in the AD dataset (GSE121212). The scatter plots showed that the expression level of CCR5 was positively correlated with Th1 (r=0.80, *P*<0.001), Th2 (r=0.66, *P*<0.001), Th17 cells (r=0.64, *P*<0.001) (**Figures 7D–F**).

### Prediction and verification of TFs

**Figure 8:**
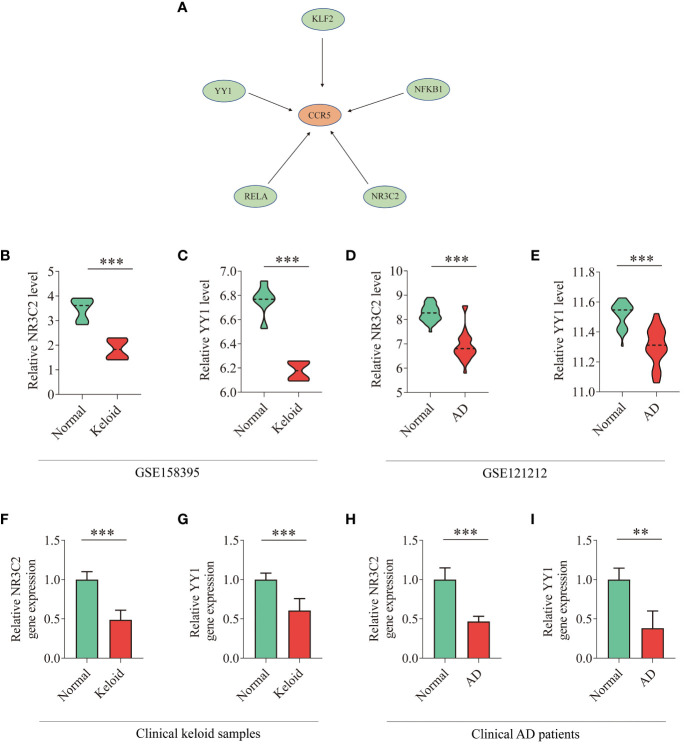
TFs regulatory network and expression levels in GEO database and clinical samples. **(A)** TFs regulatory network. TFs were marked in green color, and the key gene CCR5 was marked in red color. **(B–E)** The expression level of NR3C2 and YY1 in gene expression profiles of keloid (GSE158395) and AD (GSE121212). **(F–I)** The expression level of NR3C2 and YY1 in clinical samples of keloid and AD. T-test was used to compare the two sets of data. *P < 0.05; **P < 0.01; ***P < 0.001.

Based on the TRRUST database, we found that 5 TFs could regulate the expression of CCR5 (**Figure 8A**). Furthermore, we found that 2 TFs, NR3C2 and YY1, are less expressed in keloid and AD samples than in the control group of GSE158395 and GSE12121 (**Figures 8B–E**). The same results were confirmed in the clinical samples (**Figures 8F–I**).

## Discussion

In recent years, RNA-seq has become a powerful tool to study development and explore molecular dysregulation in disease (35, 36). In addition, integrative bioinformatics analysis and machine learning methods are applied to explore the key genes, underlying mechanisms and therapeutic targets (37). To the best of our knowledge, the present study is the first to elucidate the association between keloid and AD disease by applying a variety of comprehensive bioinformatics methods.

Keloid is an abnormal proliferation of skin connective tissue that results in the deposition of bulky collagen fibers in the dermis, and the pathogenesis of keloid has not been fully elucidated. The inflammatory hypothesis suggests that tissue injury triggers an excessive inflammatory response, with large numbers of inflammatory cells infiltrating the wound tissue (38). The infiltrating inflammatory cells then release cytokines that stimulate fibroblast activation and synthesis of large collagen fibers, leading to keloid formation. Ghazizadeh et al. first documented increased expression of IL-6 and its receptor in keloid fibroblasts, together with a large number of collagen fibers in the dermis (39). Current research suggests that T cells play a complex role in the inflammatory response and that cytokines are important mediators promoting the fibrosis process (40–42). Further studies show that the Th2 cell-associated cytokines IL-4/IL-13 and the Th17 cell-associated cytokines IL-17A/IL-22 were key drivers of fibrosis in a variety of organs, including lung, liver and kidney (43–46–). IL-4/IL-13 could stimulate transforming growth factor (TGF-β) secretion from macrophages to promote fibrosis (43). Similarly, IL-17A produced by Th17 cells could induce the recruitment and secretion of cytokines such as TGF-β by neutrophils and macrophages and regulate collagen production to promote fibrosis (44–46). Recently, RNA-seq analysis has identified multiple T helper pathways in the inflammatory milieu of keloid, including the Th2, Th1 and Th17/Th22 axes (15). These results suggest that Th cells are involved in keloid formation, but the exact mechanism of the Th cell-mediated inflammatory response leading to fibrosis is unclear.

AD is a chronic inflammatory skin disease predominantly mediated by Th2 cells. A growing body of clinical evidence suggests that patients with AD have a significantly increased incidence of keloid, and AD is the comorbidity of keloid and hypertrophic scars with statistical significance among participants in the UK Biobank. Recently, Diaz et al. reported the case of a 53-year-old African-American man with moderate to severe AD who was treated with the anti-IL-4 receptor α monoclonal antibody dupilumab (47). After 7 months of treatment, his AD condition improved significantly. At the same time, the patient’s keloids were reduced, flattened and blurred in appearance. In addition, Wong et al. reported a keloid patient receiving dupilumab therapy (48). After 3 months of treatment with dupilumab, there was no significant reduction in the size of the keloid, but the patient’s symptoms of itching and pain were significantly improved. Therefore, as suggested by previous studies, keloid has a strong association with AD.

In the first epidemiological study of the relationship between gene polymorphisms and AD, Matsusue et al. reported a significant association between a disintegrin and metalloproteinase (ADAM) 33 gene polymorphisms and AD in a comparison of 140 children with AD and 258 healthy controls (49). Subsequently, a case-control study suggested that keloid development may be associated with the ADAM33 single-nucleotide polymorphisms, comparing blood test results of 283 subjects with keloids and 290 controls (50). These results suggest that keloid and AD are closely linked at the molecular level. However, the potential factors and mechanisms underlying the association between keloid and AD are not fully understood. Therefore, the aim of this study is to investigate the common pathways and hub genes involved in the pathogenesis of keloid complicated with AD.

The common transcriptional features may provide new insights into the common molecular mechanism. In this study, GSVA analysis, GO terms annotation and KEGG enrichment analyses were used to investigate the mechanism underlying keloid and AD. According to the results of GSVA analysis, both diseases shared some inflammatory and immune pathways, including TNF signaling pathway, chemokine signaling, inflammatory response and immune response. These shared pathways could be the potential mechanisms underlying the association between keloid and AD. Next, the commonly expressed DEGs of keloid and AD were selected. GO and KEGG pathway enrichment analyses revealed that the co-expressed DEGs were significantly enriched in inflammatory and immune pathways, such as Toll-like receptor signaling, Th17 cell differentiation, NF-κB signaling pathway, cytokine-cytokine interaction, chemokine signaling pathway, TNF signaling pathway, etc. Subsequently, 20 hub genes were identified from co-expressed DEGs using the PPI network. The key genes were screened from the 20 hub genes using LASSO and SVM-RFE machine learning algorithms. Finally, CCR5 was selected as the key gene with ideal performance. The immune infiltration analysis further confirmed that keloid and AD may have overlapping inflammatory pathways, such as Th1, Th2 and Th17 pathways. The key gene CCR5 was significantly and positively correlated with Th1, Th2 and Th17 cells, suggesting that the CCR5 gene is associated with the Th cell axis. Furthermore, ROC curve analysis showed that the CCR5 gene had good discriminatory power. External validation of validation datasets and clinical samples confirmed that the upregulated CCR5 expression was consistent with the discovery datasets. In addition, we also analyzed TFs related to CCR5 and verified their expression levels in the training dataset and clinical samples. The CCR5 gene played a key role and was a sensitive biomarker in the development of both keloid and AD.

CCR5 is a seven-transmembrane G protein-coupled receptor that regulates trafficking and effector functions of memory/effector T lymphocytes, macrophages and immature dendritic cells (51). It has been reported that CCR5 and its chemokine ligand (CCL5) play a critical role in hepatic and lung fibrogsis, promoting hepatic stellate cells and fibroblasts to secrete cytokines and chemokines that contribute to the proinflammatory and profibrotic milieu (52, 53). Anti-CCR2/CCR5 drugs (NCT02217475; NCT03028740; NCT03059446; NCT02330549) for liver fibrosis and non-alcoholic steatohepatitis are in phase 2 or 3 clinical trials for antifibrotic therapy (16). CCR5 may also play an important role in atopic dermatitis, and expression of CCR5 on langerin-negative CD1a^+^ DCs was characteristic for acute AD (54). Research has shown that CCR5 may promote the orchestration of eosinophil infiltration in the ongoing chronic inflammation of AD disease and may also reflect the severity of the disease (55). CCR5 is a potential drug target for a wide range of immune disorders (56).

Although previous studies have examined the hub genes associated with keloid and AD separately, few studies have explored the common molecular mechanism between them using advanced bioinformatics methods (57, 58). Due to the close association between keloid and AD, we explored and identified the common DEGs, enriched pathways, key genes and correlationship with infiltrated immune cells between the two diseases, which helped to further elucidate the mechanism of keloid and AD. The above studies suggest that CCR5 may be an important mediator of the inflammatory response and fibrotic process, and may play a pivotal role in the association linking keloid and AD. We aim to broaden the horizons of the molecular mechanisms of keloid and provide novel therapeutic targets for clinical management.

However, there are several limitations in our research. Firstly, the datasets analyzed in this study were from public GEO databases based on different platforms, which could not be directly compared. Secondly, some datasets have small clinical samples. The relevant clinical information in the datasets is not complete, such as missing disease duration, treatment history and skin lesion sites, etc. Thirdly, the function of the key gene CCR5 and its upstream/downstream pathways in keloid and AD need to be further verified, which will be the focus of our future work.

## Conclusions

In conclusion, keloid and AD share some common inflammatory and immune pathways, including TNF signaling, Toll-like receptor signaling, Th17 cell differentiation, NF-κB signaling, cytokine-cytokine interaction, and chemokine signaling pathways. The CCR5 gene was selected as the key gene that links the association between keloid and AD. Further experimental validation is required to verify the role of CCR5 in keloid with AD. This study provides a new perspective on the underlying mechanism linking keloid with AD and provides new research clues for the treatment target of keloid and AD.

## Data availability statement

The original contributions presented in the study are included in the article/**Supplementary Material**. Further inquiries can be directed to the corresponding authors.

## Ethics statement

The studies involving humans were approved by The Ethics Committee of Wuhan Union Hospital, Huazhong University of Science and Technology. The studies were conducted in accordance with the local legislation and institutional requirements. The participants provided their written informed consent to participate in this study.

## Author contributions

BZ: Conceptualization, Methodology, Software, Writing – original draft. NZ: Investigation, Validation, Writing – original draft. YL: Investigation, Writing – review & editing. ED: Validation, Writing – review & editing. LP: Data curation, Writing – review & editing. YW: Validation, Writing – review & editing. LY: Writing – review & editing. HS: Supervision, Validation, Writing – review & editing. JT: Funding acquisition, Project administration, Supervision, Writing – review & editing.

## Supplementary material

Supplementary Figure 1 Analysis of immune cell infiltration in the keloid and AD validation datasets. **(A)** The relative percentage of 28 immune cells in each sample of the keloid dataset (GSE188952). **(B)** The relative percentage of 28 immune cells in each sample of the AD dataset (GSE32924).

Supplementary Figure 2
**(A, B)** The levels of factors (IFNγ, IL4, IL13, IL17A, IL22, ADAM3) related to Th cells in clinical keloid and AD samples.
