# Supplementary material for: Identification and validation of CCR5 linking keloid with atopic dermatitis through comprehensive bioinformatics analysis and machine learning
Source: Front Immunol. 2024 Feb 27;15:1309992. doi: 10.3389/fimmu.2024.1309992 (PMC10927814; doi:10.3389/fimmu.2024.1309992)
Supplement: Supplementary Figure 1 — Analysis of immune cell infiltration in the keloid and AD validation datasets. (A) The relative percentage of 28 immune cells in each sample of the keloid dataset (GSE188952). (B) The relative percentage of 28 immune cells in each sample of the AD dataset (GSE32924). [file DataSheet_1.docx]

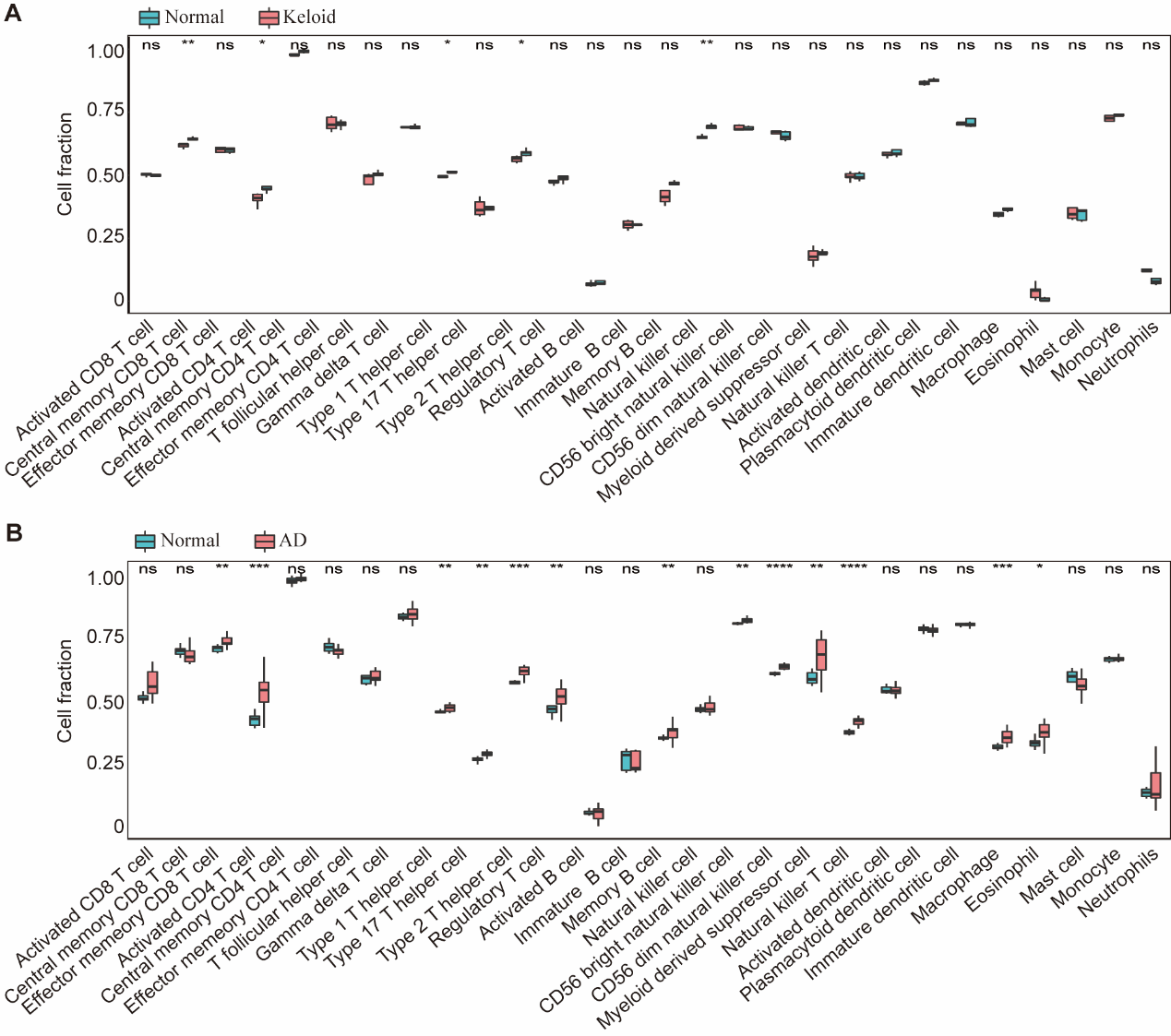


**Supplementary Figure 1. Analysis of immune cell infiltration in the keloid and AD validation datasets.** **(A)** The relative percentage of 28 immune cells in each sample of the keloid dataset (GSE188952). **(B)** The relative percentage of 28 immune cells in each sample of the AD dataset (GSE32924). * *P* < 0.05, ** *P* < 0.01, *** *P* < 0.001. ns: no significance.


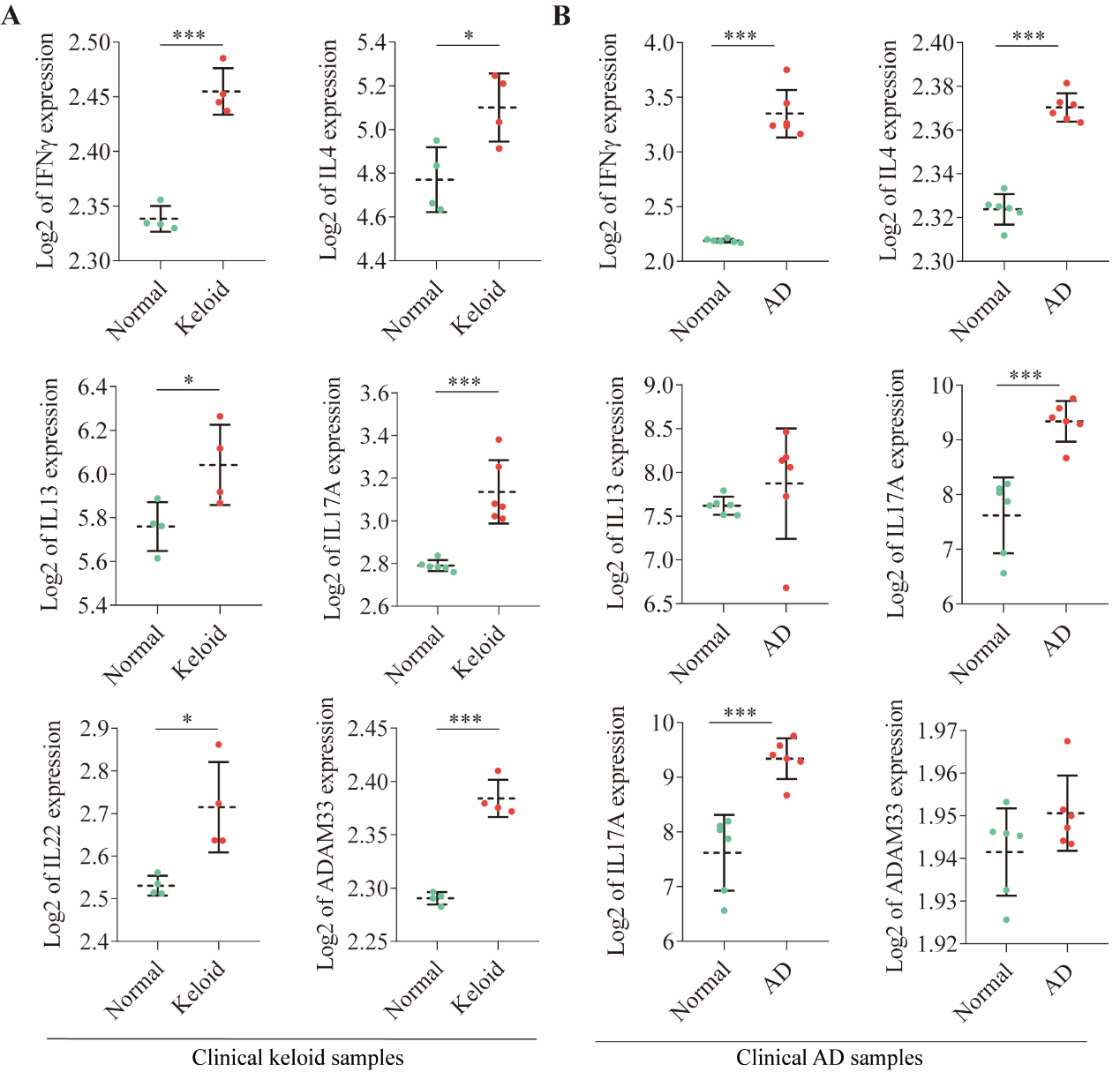


**Supplementary Figure 2.** (A-B)The levels of factors (IFNγ, IL4, IL13, IL17A, IL22, ADAM3) related to Th cells in clinical keloid and AD samples. * *P* < 0.05, ** *P* < 0.01, *** *P* < 0.001.
